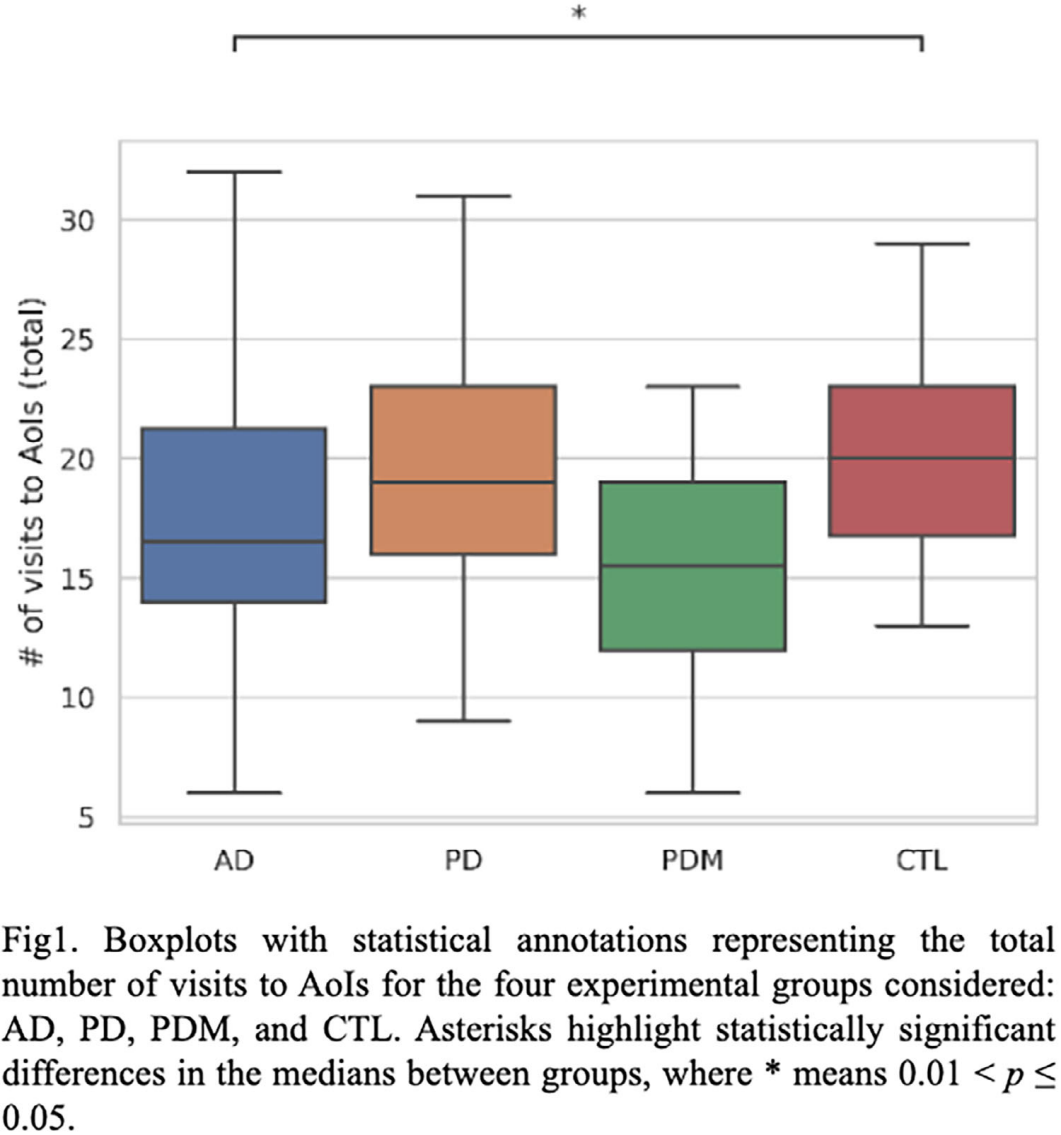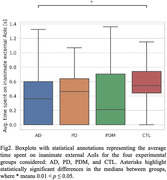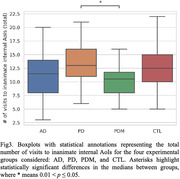# Analyzing Attention Focus in the Cookie TheftPicture Description Task Using Word Alignment

**DOI:** 10.1002/alz.091687

**Published:** 2025-01-09

**Authors:** Anna Favaro, Thomas Thebaud, Najim Dehak, Ankur Butala, Esther S Oh, Laureano Moro‐Velazquez

**Affiliations:** ^1^ Johns Hopkins University, Baltimore, MD USA; ^2^ Johns Hopkins University School of Medicine, Baltimore, MD USA

## Abstract

**Background:**

Visuospatial problems are one of the possible signs of Alzheimer’s Disease (AD). Notably, various studies on eye movement in static image searches reveal that individuals with dementia fixate on fewer Areas of Interest (AoIs) for a significantly shorter duration than normal controls (CTLs). Building on prior works on speech and eye movement, in this study we examined word timestamps to infer each subject’s focus of attention while describing the Cookie Theft picture. Namely, we computed various interpretable features encoding attention focus and compared them across different neurological disorders.

**Method:**

Cookie Theft Picture description tasks were recorded from subjects with AD (n=20), Parkinson’s Disease (PD) (n=43), Parkinson’s disease mimics (PDM) (n=16), and CTLs (n=33). First, speech transcriptions and word timestamps were generated using WhisperX automatic speech recognizer. The following features were then computed using word timestamps: time spent in the AoIs, time to approach the first AoI, number of visits to the AoIs, transition time from one AoI to another, and time elapsed between the first and last AoI mentioned. Features were computed considering all the animate AoIs, all the inanimate AoIs, and all AoIs collectively. Completed the feature extraction, non‐parametric pair‐wise Mann‐Whitney U tests of significance were conducted to determine whether there were significant differences between experimental groups for each feature. To control the False Discovery Rate, we applied the Benjamini–Hochberg correction.

**Result:**

Results from the statistical analysis showed that subjects with AD reported a significantly fewer number of visits to the AoIs and a shorter amount of time spent on inanimate external AoIs than CTLs. The AD group also reported a greater variability in the transition time from one AoI to another. Moreover, the PDM group spent significantly more time (on average) than the PD group on animate AoIs but reported a fewer number of visits to inanimate AoIs located internally to the house than PDs.

**Conclusion:**

Our preliminary analysis confirmed the difficulties of subjects with cognitive decline to voluntarily direct attention to central cues and maintain attention focus on given AoIs. Our future work will include eye‐tracking technology to analyze the relationship between attention focus from speech and eye movement.